# Using Intersectionality to Identify Gendered Barriers to Health-Seeking for Febrile Illness in Agro-Pastoralist Settings in Tanzania

**DOI:** 10.3389/fgwh.2021.746402

**Published:** 2022-01-28

**Authors:** Violet Barasa, Jennika Virhia

**Affiliations:** ^1^The Institute of Development Studies, University of Sussex, Brighton, United Kingdom; ^2^The Institute of Health and Wellbeing, School of Social and Political Science, The University of Glasgow, Glasgow, United Kingdom

**Keywords:** gender, health seeking behavior, febrile illness, zoonoses, Tanzania, East Africa, fever, intersectionality

## Abstract

**Background:**

Research has shown that gender is a significant determinant of health-seeking behavior around the world. Gender power relations and lay etiologies of illness can influence the distribution of household resources, including for healthcare. In some rural settings in Africa, gender intersects with multiple forms of health inequities, from proximal socio-cultural factors to more “upstream” or distal health system determinants which can amplify barriers to health-seeking for specific groups in specific contexts.

**Aim:**

We used an intersectionality approach to determine how women in particular, experience gendered barriers to accessing healthcare among Maa and non-Maa speaking agro-pastoralists in northern Tanzania. We also explored lay etiologies of febrile illness, perceptions of health providers and rural health-seeking behavior in order to identify the most common barriers to accessing healthcare in these settings.

**Methods:**

Mixed method ethnographic approaches were used to collect data between 2016 and 2018 from four Maa-speaking and two Swahili-speaking agro-pastoralist villages in northern Tanzania. Maa-speaking villages were based in Naiti, Monduli district while non-Maa speaking villages were selected from Msitu in Babati district. Data on health seeking behaviors was collected through semi-structured questionnaires, in-depth interviews, focus group discussions, and home and facility-based participant observation.

**Findings:**

The results primarily focus on the qualitative outcomes of both studies. We found that febrile illness was locally categorized across a spectrum of severity ranging from normal and expected illness to serious illness that required hospital treatment. Remedial actions taken to treat febrile illness included attending local health facilities, obtaining medicines from drug sellers and use of herbal remedies. We found barriers to health-seeking played out at different scales, from the health system, community (inter-household decision making) and household (intra-household decision making). Gender-based barriers at the household had a profound effect on health-seeking. Younger married women delayed seeking healthcare the most, as they often had to negotiate health-seeking with husbands and extended family members, including co-wives and mothers-in-law who make the majority of health-related decisions.

**Conclusion:**

An intersectional approach enabled us to gain a nuanced understanding of determinants of health-seeking behavior beyond the commonly assumed barriers such lack of public health infrastructure. We propose tapping into the potential of senior older women involved in local therapy-management groups, to explore gender-transformative approaches to health-seeking, including tackling gender-based barriers at the community level. While these social factors are important, ultimately, improving the public health infrastructure in these settings is a first step toward addressing structural determinants of treatment-seeking.

## Introduction

Zoonotic-associated febrile illness present a significant health burden for both people and livestock in herder populations in northern Tanzania (1–3). This is attributed to the daily interaction that people have with their livestock, including husbandry practices, and to the habitat overlaps between people, livestock as well as wildlife, from which cross-transmission of fever-causing zoonoses is possible (4, 5). In addition, a number of studies have highlighted the prevalence of many zoonotic infections which are endemic in pastoralist and agro-pastoralist settings in northern Tanzania, including brucellosis (1, 3, 6, 7), anthrax (8, 9), Rift Valley fever (10), bovine tuberculosis (11), and rabies (12). These diseases affect people's health and productivity, with significant implications for livelihoods (13–15).

Moreover, exposure to zoonotic risks is determined by socio-cultural norms around division of labor, and it depends on what people do, with what animals, for how long and where (16), which results in unequal distribution of risk. It is often the poorest people, mainly women and girls, who come into contact with zoonotic pathogens because of their roles as foragers of food, fuel wood and medicine from bushes which vectors inhabit (17–19). Women and girls are also more likely to care for sick stock in many livestock-keeping communities, and therefore are more likely to come into direct contact with contaminated animal fluids and aesorols. For example, Dzingirai et al. (17) found that women and girls from poor households in Zimbabwe are more at risk of trypanosomiasis due to their foraging in forests where they come into contact with infected Tsetse flies.

In Tanzania, gender plays an important role in shaping the way responsibilities around livestock are allocated in pastoralist and agro-pastoralist communities (20, 21). In this case, gender determines different types of exposure to animals, and arguably susceptibility to zoonoses, as certain individuals and groups may be more exposed to sick livestock than others.

However, it is the intersection or interaction of gender and the various social, economic and political barriers that produce varying levels of health inequalities and exclusion for specific individuals or groups in a particular setting. These intersections are often underappreciated in health research. As Larson et al. (22) argue, using intersectional approaches enhances our understanding of inequality by enabling reflection on the complexity of the world and by showing how social stratifiers such as gender, age, location etc., are “mutually constituting and intersecting in dynamic and interactive ways” (p. 965). For example, while people living in remote, hard to reach areas may all experience health exclusion at a structural level (such as lack of access to clinics, electricity, water, laboratory services etc.), it is often the case that certain groups experience multiple layers of marginalization, which amplify their exclusion from health services. For instance, while women in many countries face discrimination on account of their gender, it is also their ethnicity, socio-economic class, age, marital status, and location that interact to shape their lived experiences of marginalization, even where structural barriers are reduced or minimized (23). Furthermore, while the specific intersections that determine marginalization vary by country and context, Kabeer (24) points out that it is the interaction of a person's identities (for example, being female, old, poor, disabled, etc.) that gives rise to the most pronounced forms of discrimination (24).

For agro-pastoralist communities, a repertoire of marginalisations exacerbate the challenge of achieving optimal health. Informal social relations factors and gender (25) are interwoven with broader political drivers such as exclusion from policy processes, non-existent or severely constrained health services (26) among other factors. Following McCollum et al. (27) who used intersectionality to explore how devolution in Kenya created additional layers of health exclusion for vulnerable people, this paper explores how health barriers and challenges are experienced uniquely by different groups and individuals as a result of multiple and converging identities. We aim to highlight the most important barriers to accessing health in our study areas, and propose policy insights to help remedy this.

The remainder of this paper proceeds as follows.

After a brief overview of the health landscape in Tanzania, we introduce the study sites and describe the materials and methods used for our studies. We draw on the data to examine core themes: first on lay etiologies of febrile illness in humans and animals, and secondly on health-system and socio-cultural barriers that shape agro-pastoralist experiences with health. This is followed by a discussion on health inequalities at the intersection of culture and structure. The paper concludes by proposing ways of reducing gender inequalities in access to health, and we agree with Workman et al.,'s call to put “cultural information first, rather than using it as an explanatory feature for issues/risks within a project” [(28), p. 411].

### Healthcare Landscape in Tanzania

Healthcare coverage in Tanzania, like in many LMICs, is limited due to a lack of resources available to support health systems. Leonard (29) and Mbugi et al. (30) highlight that major limiting factors for both human and veterinary health coverage in Tanzania, include, the extremely limited resource base and a shortage of qualified personnel that have resulted in a huge burden of disease. These authors estimate that at least 60% of the population in Tanzania do not have access to formal healthcare services and rely on traditional and alternative healthcare systems to meet their day-to-day health needs [see also Tarimo (4), Seth et al. (5), Munga and Maestad (31), and Marsland (32)]. Critical staff shortages are a major challenge for access to healthcare. On average, there are 1.4 health workers per 10,000 people in Tanzania, 10 times higher than the WHO recommended doctor to patient ratio, and this number varies greatly between districts (29, 31, 33). These authors highlight that the situation is worse in semi-arid areas, which are typically inhabited by pastoralists and agro-pastoralists in the north of the country. These areas have the fewest skilled medical personnel [just eight per cent of all health workers per capita (31, 33, 34)].

These inadequacies within the health system have driven both supply and demand for informal health providers across Africa. In many cases, traditional and/or informal therapies interact and even hybridize with biomedicine to create what Lock and Nguyen [(35), p. 55] term “diverse therapeutic economies.” The adoption of traditional treatment has also been attributed to lay perception of health risks and etiologies of illness that are negatively associated with seeking help from trained allopathic doctors (36).

Yet, even in formal health centers in these areas, there are no clear guidelines on the management of health problems like febrile illness (5, 33, 37, 38). For example, in their study of febrile patients admitted to a hospital in Morogoro in Tanzania, Seth et al. (5) found that clinicians arrived at presumptive diagnoses as they lacked appropriate tests to help them accurately diagnose febrile illness in patients after negative malaria testing. Indeed, many health centers only have a malaria rapid diagnostic test (mRDT) and lack laboratory capacity to diagnose non-malarial febrile illness (5, 38–40). Although official government reports indicate that malaria has decreased in Tanzania (4, 38, 41), over-diagnosis of malaria is common. A study by Crump et al. (3) on pediatric and adult hospital admissions in Moshi, northern Tanzania, found that although over 60 per cent of patients had received a malaria diagnosis from the clinicians, in roughly 26% of cases the actual cause of their febrile illness was zoonotic (including brucellosis, leptospirosis and Q-fever). Similarly, Chandler et al. (42) found that many clinicians in Tanzania readily diagnosed febrile patients with malaria, but they did not consider zoonotic causes as they were not aware of these diseases [see also Chipwaza et al. (43) on overdiagnosis of malaria in Tanzania].

Additionally, much of the health research in Tanzania focuses on health facility-based barriers to accessing healthcare, with little attention on other socio-cultural barriers or how gender power relations influences access to healthcare (23, 24), particularly for populations that are exposed to zoonotic disease such as in northern Tanzania [see for example Leonard (29), Kruk and Mbaruku (44), and Mubyazi et al. (45) on facility-based barriers to health-seeking].

Moreover, lay perceptions of fever and its causes in pastoralist and agro-pastoralist settings in Tanzania remain understudied (46). Research in this area has tended to focus on lay understandings of the biomedical disease malaria; for example the ability of mothers to recognize malarial symptoms in their children (4, 47). These studies have, not surprisingly, given the Swahili/indigenous classification of serious fever as malaria, revealing widespread recognition of malaria symptoms amongst Tanzanians, who perceive it to be the most important cause of fever and who, in many cases, use the terms “fever” and “malaria” interchangeably [see also Seth et al. (5), and Hertz et al. (46)].

Finally, there is a dearth of empirical data on determinants of health-seeking behavior for febrile illness in agro-pastoralist settings, in particular, studies that use a gender lens and innovative methodologies. Even less so, is research on the intersection between structural, community, and household-level barriers and how they affect different groups in different ways, and what can be done to mitigate against these barriers. This paper therefore, makes both empirical, methodological and theoretical contributions to this area of work, by employing an intersectionality approach that is seldom used in health research, as well as using an ethnographic methodology. The paper also proposes some practical ways of tackling gender-based barriers to health-seeking in agro-pastoral settings.

### Case Study: Naiti and Msitu Villages in Northern Tanzania

This paper draws from combined findings of studies on health-seeking behavior for febrile illness, which took place in two agro-pastoral villages in northern Tanzania (Naiti—Maa-speaking and Msitu—Swahili speaking).

The studies were part of two independent PhD studies carried out concurrently between 2016 and 2018. The former study was conducted by Barasa (20) (hereafter VB) while the latter by Virhia (48) (hereafter JV). A distinction is made between Maa and Swahili-speaking communities to emphasize the slight socio-cultural variations and the impact this may have on health-seeking behavior. Both studies investigated patterns of health-seeking behavior in livestock-keeping households, and common barriers to accessing timely healthcare were explored in depth. JV's study focussed on broader political-economic, institutional and structural barriers, including facility-based factors while VB's focussed more on micro and community-level barriers, including gender, age and other social stratifiers. Thus, the two studies complement each other by highlighting the scales at which health-seeking plays out in agro-pastoral settings, from broad scale structural and institutional barriers (JV) to intra and inter household dynamics (VB). The results were analyzed independently, using thematic analysis and comparisons were drawn to show converging and diverging themes. The combined findings are presented later in the paper, and are juxtaposed with the literature on health systems and health-seeking behavior in these settings.

We describe the study studies below.

### Naiti Village

**Figure 1:**
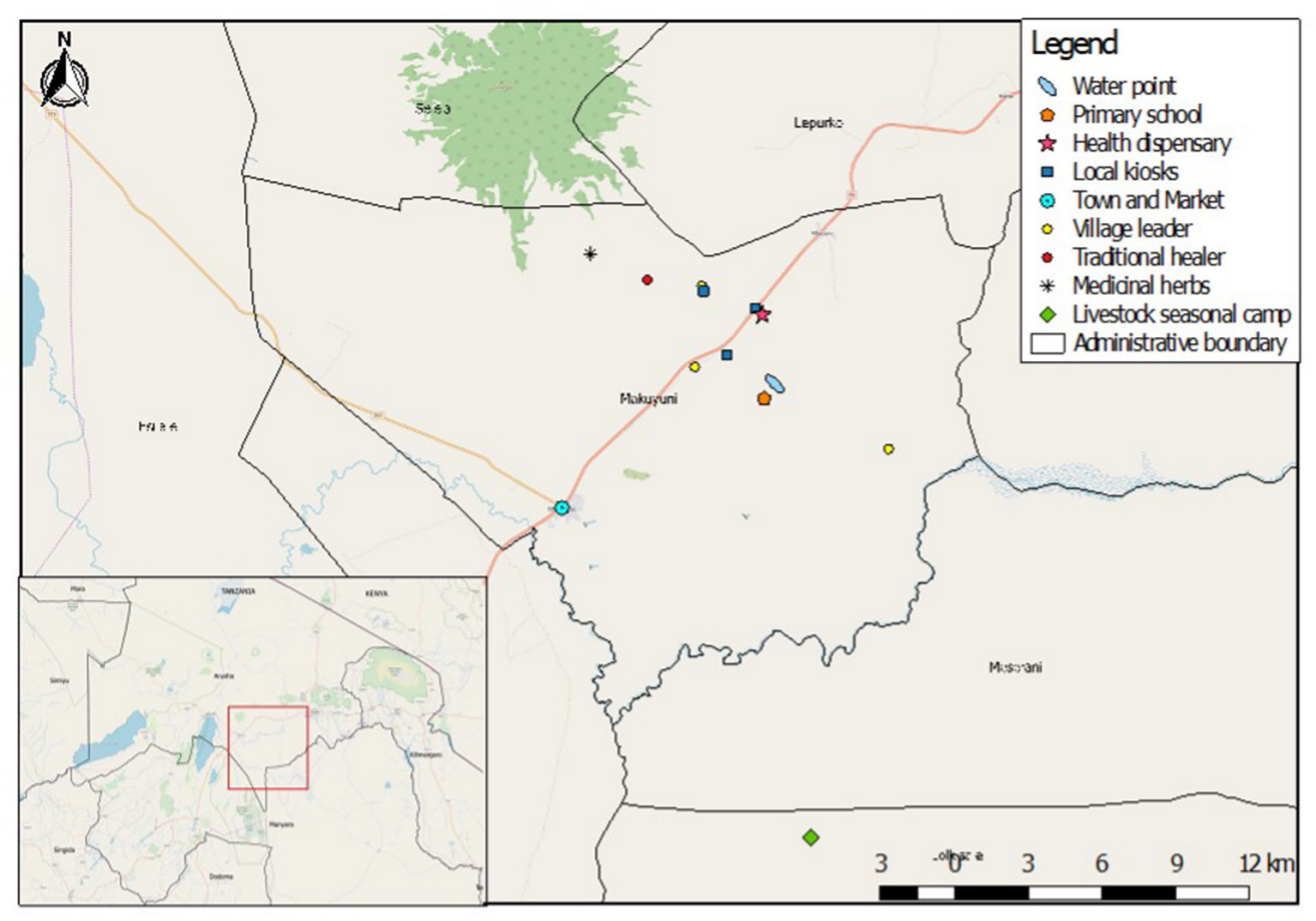
Map of Naiti village with markings indicating important places.

Naiti village lies within the Maasai Steppe (49). It is an area of vast savannah plains located along the Arusha-Manyara road in Makuyuni ward, Monduli district, northern Tanzania (Figure 1). Its inhabitants can be best described as Maa-speaking agro-pastoralists, that is, in addition to livestock production, they are also involved in crop cultivation (50–52). Most people here “own” small plots of land where they grow food crops and raise their livestock and are not wholly transhumant (the practice of moving herds through seasons). Land “ownership” here is contentious as the land is public property and no individual title deeds are held.

### Msitu Village

**Figure 2:**
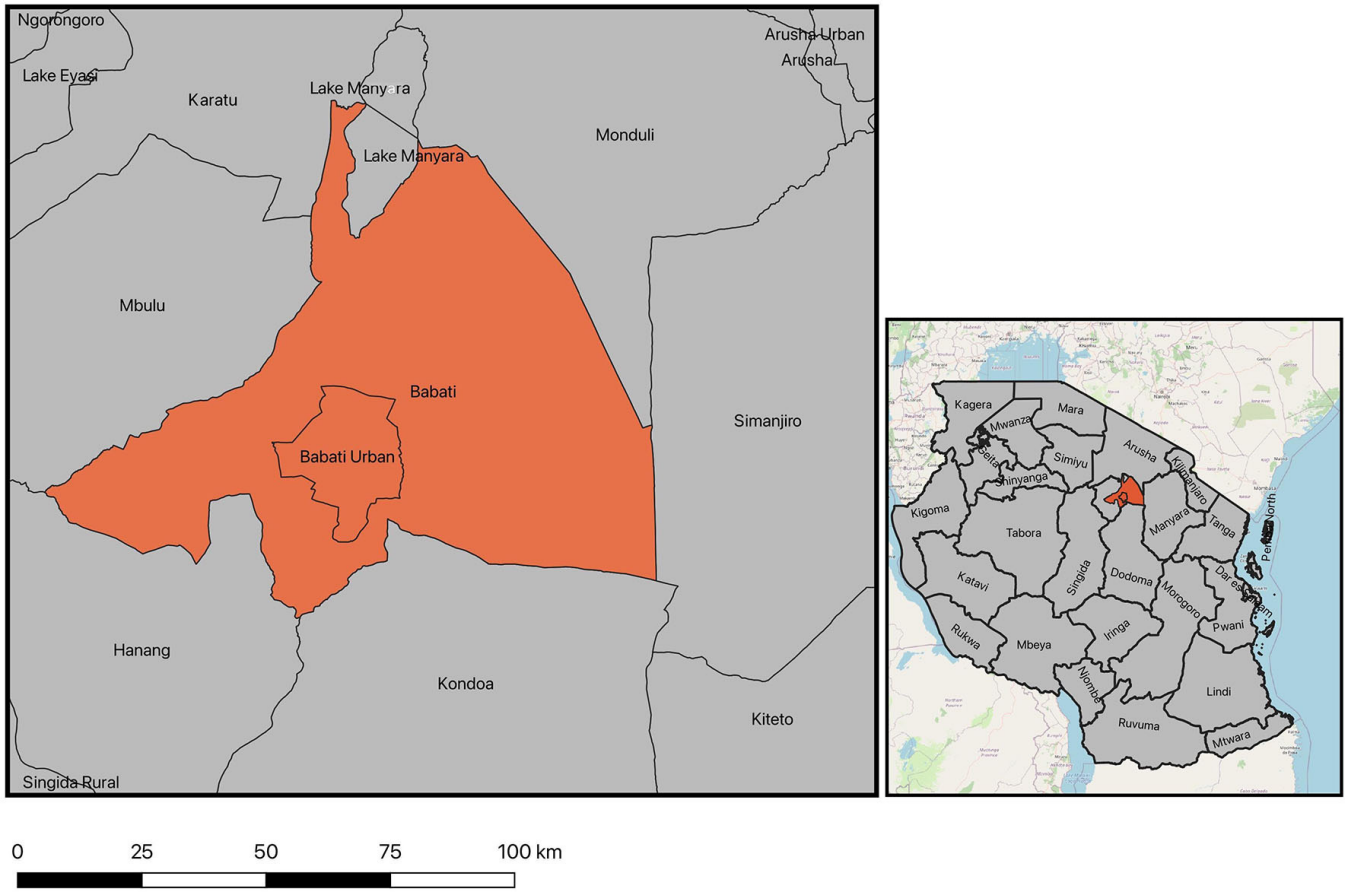
Map of Babati district encompassing Msitu village. Source: (48).

Msitu village is located in the Babati district of Manyara region in northern Tanzania (Figure 2). Its inhabitants belong to a mix of tribal identities, primarily consisting of Iraqw and Nyaturu groups, in addition to some Arusha and Barbaig families. Like Naiti, Msitu residents are traditionally agro-pastoralists where they own a small amount of land on which they grow maize, peas and beans for subsistence (or selling in times of need) and raise livestock to support farm-based livelihoods.

As we described earlier, livestock-keeping communities live in some of the most remote and hard to reach areas in Tanzania, characterized by poor public health infrastructure that result in limited healthcare options for many families. At the time of this study, there was only one basic health dispensary in Naiti village, which was established in 2014. The clinic was staffed with one clinical officer. The training of a clinical officer is very basic, often taking no more than 3 years of training in basic medical skills. He was responsible for a population of about 3,000 people spread across a vast area, with poor infrastructure making it difficult for many to access the dispensary. Similarly, Msitu village had a dispensary which provided primary care to children and elderly. In both settings the clinics were operating under severe constraints as they lacked medicines, diagnostic equipment, staffing, electricity and water.

**Figure 3:**
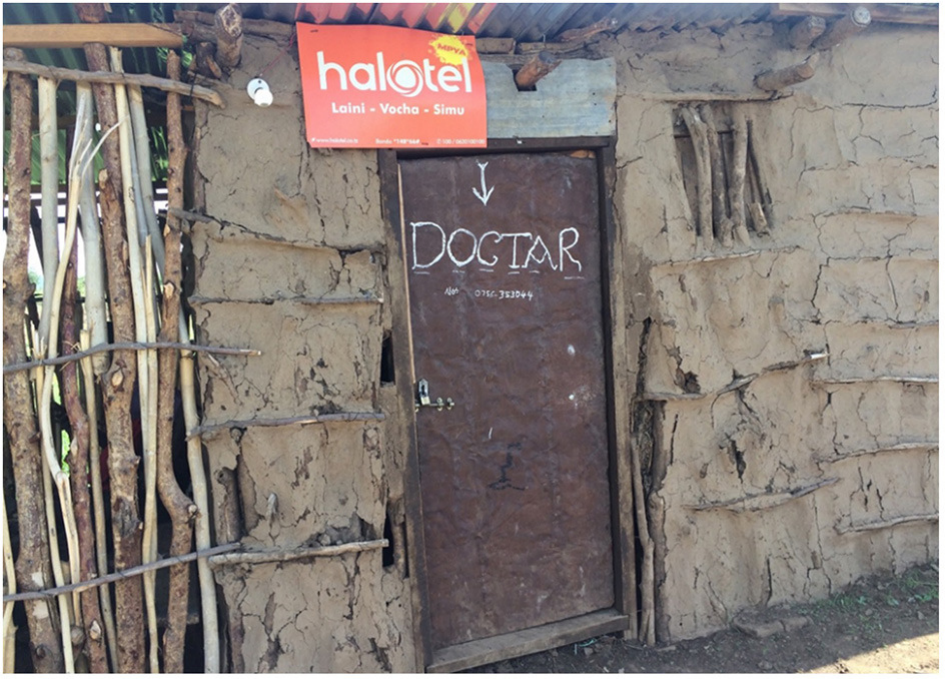
A basic shop or “duka” in Naiti village where locals obtain most of their medicines.

Consequently, for most of their health problems, residents of Naiti and Msitu rely on a range of formal and informal providers who fill the gap of unmet demand in healthcare. These include traditional healers and drug peddlers, who flourish but are not well-regulated by the government. In Naiti, families routinely purchase medicine from a local kiosk (Figure 3), locally referred to as *duka* or shop—a basic structure that sells unlabelled drugs that come with no packaging, alongside other everyday household necessities like soap, salt and paraffin for cooking and lighting. In an interview with the seller, who is fondly referred to as *dokta* for doctor, he described the most commonly purchased drugs as paracetamol and penicillin, which he said were used in treating the common complaints of his “patients,” including perceived febrile illness.

**Figure 4:**
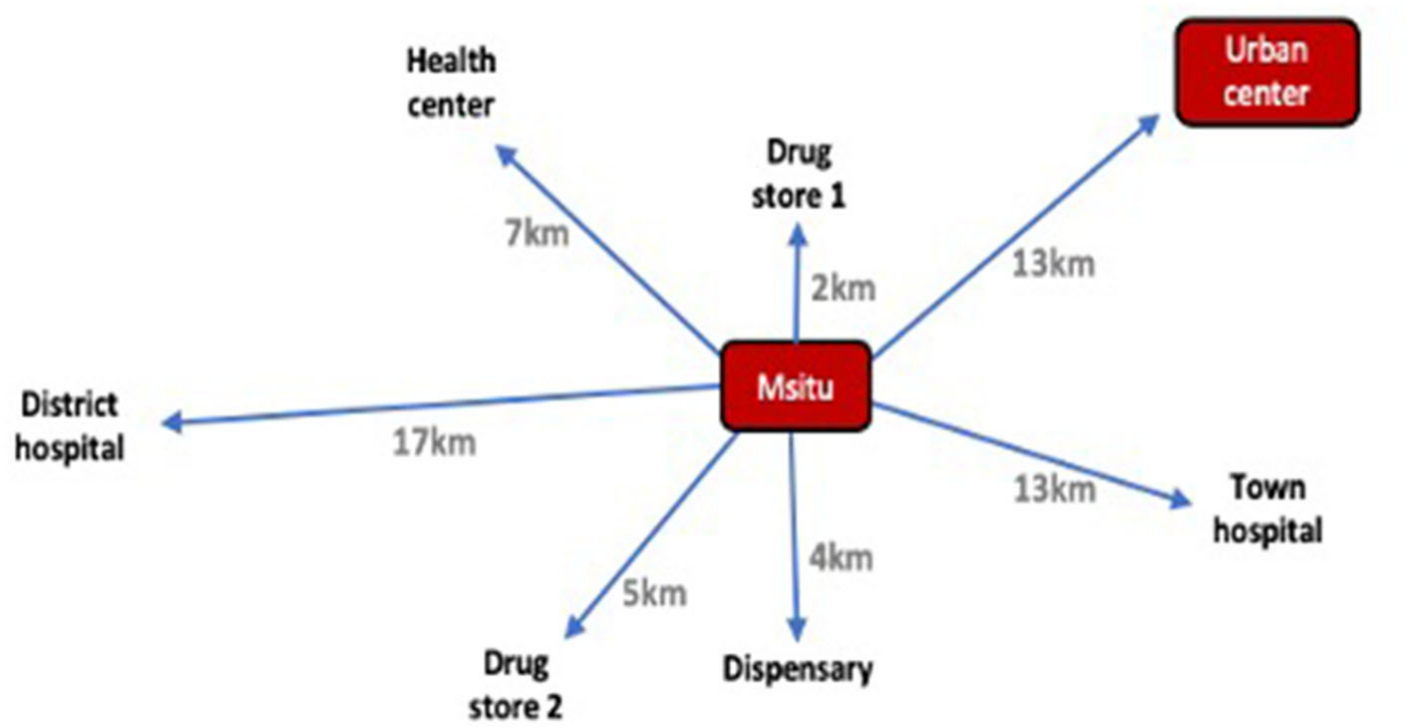
Distribution of healthcare facilities in relation to Msitu village.

In Msitu, families most commonly reported buying medication from two designated drug stores (*duka la dawa*s) which sold a range of over the counter drugs in addition to a selection of broad spectrum antibiotics (such as amoxicillin). Drug store vendors in Msitu had some experience and training in dispensing medication. An example of the distribution of health facilities in Msitu is shown in Figure 4.

## Materials and Methods

While there were methodological differences between the two studies, both were grounded in ethnography as a core methodology. Both authors lived in their respective study areas throughout the duration of fieldwork, collecting data relating to etiologies and health-related behaviors for fever. All data was collected by the authors (VB, JV) who were each assisted by two researchers (one male and one female for each). Assistants were recruited from within the local communities and assisted with translations from English to Kiswahili and Maa, the two local languages commonly used. We each used purposive (VB, JV) and random (JV) sampling to recruit a total of 479 people (255 women and 224 men) to take part in the study. Sample size was capped at 479 when data saturation was reached and no new themes emerged from the data. Eligibility criteria included belonging to an agro-pastoralist household, having lived in the village for more than 10 years and being 18 years or over. We made efforts to recruit across the socio-economic and gender spectrum, including men, women, younger, older, abled, disabled and elderly. Respondent characteristics for each study site are outlined in Tables 1, 2.

**Table 1:** Socio-demographic features of participating households in Naiti.

**Wealth category**	**Count**	**Average H/hold size**	**Gender**		**Average education**	**Livestock ownership**				**Land (hectares)**	**Other assets**
			**Male**	**female**		**Cattle**	**Small stock**	**Donkey**	**Poultry**		
Better off	14	34	12	22	5 members: secondary others: basic	21	80	3	50	6–10	3 mobiles, 1 radio, 1 motorcycle
Poor	126	26	9	17	Basic	12	30	2	30	3–6	2 mobiles
Very poor	41	20	6	14	Basic or nil	3	15	0	20	2–3	1 mobile
Poorest	19	15	4	11	Nil	1	12	0	12	1–0	Nil

**Table 2:** Respondent characteristics in Msitu.

**Characteristics**	**Ziwa (subvillage)**	**Mlima (subvillage)**	**Total (*n* = 100)**
**Gender**			
Male	20.0	36.0	56.0
Female	27.0	17.0	44.0
**Age groups**			
18–24	0.0	1.0	1.0
25–34	9.0	6.0	15.0
35–44	13.0	10.0	23.0
45–54	11.0	13.0	24.0
55–64	9.0	10.0	19.0
65–74	6.0	8.0	14.0
75+	1.0	1.0	2.0
Unknown	1.0	1.0	2.0
**Education**			
Primary	35.0	44.0	79.0
Secondary	0.0	4.0	4.0
Post-secondary	1.0	1.0	2.0
No education	5.0	10.0	15.0
**Family size**			
1–5	22	18	40
6–10	26	32	58
11–15	2	0	2
**Tribe/ethnicity**			
Nyaturu	27	6	33
Iraqw	13	38	51
Barabaig	1	3	4
Arusha	4	0	4
Other	4	3	8

We each used a chronological, qualitatively driven sequential design (53) which began with the administration of surveys and concluded with qualitative data collection. The field teams first administered a survey questionnaire to all eligible households, and collected quantitative data on demographic variables, febrile illness episodes in the household, interpretation of symptoms of illness, its causes and treatment options pursued, and on perceived most important barriers to treatment seeking. Survey data was then used to inform further points of inquiry for the qualitative work, which included recasting and further developing study questions. Survey data also allowed us to expand the range of perspectives included, as well as provide a sampling framework for identifying participants for the qualitative aspects of the study. We analyzed survey results qualitatively using thematic coding, and followed up on emergent themes with in-depth interviews and focus groups regarding recent experiences with fever. While both studies employed a quantitative component for initiation, namely, the survey technique, qualitative methods were the dominant data collection tools. As a result, this paper primarily focuses on the qualitative outcomes which provide richness of detail on respondents' etiologies of febrile illness, as well as their experiences, worldviews and concerns when seeking treatment. The qualitative aspects of data collection are discussed further below.

Interviews were conducted with 66 individuals (23 women and 24 men), as well as 43 key informant interviews with 17 men and nine women. Those who participated in interviews included: lay healers, community elders, local leaders, medical professionals and animal health workers. Lay healers provided information on local therapies and emic interpretations of febrile illness and healing. In keeping with our intersectional approach, we endeavored to interview participants across a range of social stratifiers to probe how they uniquely face multiple forms of disadvantage regarding febrile illness. Interviews were conducted longitudinally with the same cohort of people across a 10-month period.

Focussed group discussions (FGDs) on the convergent and divergent themes that emerged from the survey and the interviews were held with different categories of study participants (such as men and women, leaders and non-leaders), in groups of no more than 10, and this helped to clarify the extent and quality of findings produced by the survey and individual interviews. FGDs were conducted in such a way to allow all participants to speak freely about their experiences with febrile illness. Facilitators took extra steps to note if particular respondents were quiet or overlooked, ensuring that all voices of participants were respected and heard throughout the discussions. In Maa-speaking communities where mixed gender gatherings are uncommon participants were disaggregated by sex and gender, as was the analysis of the FGDs.

Qualitative data including focus group discussions were analyzed using thematic analysis and were scrutinized for converging and diverging themes. In VB's sudy these were then clustered into core thematic areas, which included a livelihood activity profile of each participant for a typical day, patterns of human-animal interaction, and their framing of zoonoses and risk. Participants were asked to rank febrile illnesses from the most to the least commonly experienced in their households. Lists were clustered per household (where more than one adult member of the household responded to the questionnaire) and household-level responses were compared against variables such as gender roles, age, wealth status, distance to health center, education, and access to information technology such as mobile phone and radio. In both studies participants were probed for knowledge and experience of febrile symptoms, perceived causality, the sequence of remedial actions taken to treat fever and, finally, their evaluation of these treatments and health providers. Open-ended survey data was triangulated with interview and focus group data which provided us with a detailed and holistic picture of participants' experiences with fever in both settings.

### Ethics

All participants provided written or verbal informed consent. The study was approved by the Ethics Review Committees of the Kilimanjaro Christian Medical Center and National Institute of Medical Research in Tanzania, and in the UK by the Ethics Review Committees of the College of Medical, Veterinary and Life Sciences at the University of Glasgow and the Institute of Development Studies at the University of Sussex. Approval for study activities was also provided by the Tanzanian Commission for Science and Technology (COSTECH) and by the Tanzania Wildlife Research Institute (TAWIRI) as well as by regional, district, ward and village-level authorities in the study area. All individual names, including any identifying information have been changed in order to preserve anonymity. Some place names have also been changed, except in mobile pastoralist study sites with no particular territorial boundaries.

### Results: Scalar Influences on Health-Seeking Behavior

We used an intersectionality approach to understand the simultaneous inequalities that communities living in resource-limited settings grapple with, and how these converge to create some of the most enduring barriers to accessing healthcare. Many anthropological studies on health and health-seeking fail to incorporate what Chambers [(54), p. 29] refers to as “clusters of disadvantage” or intersecting disadvantages in health research. These tend to focus on one thing or the other, for example on how gender influences who gets sick and who does not (17–19), or how treatment seeking is approached. Yet, as we discussed earlier, intersectionality can enable researchers to explore how different social, economic and political domains intersect to produce varying levels of power and privilege for individuals or groups in a particular setting (27) and at different scales.

This approach allowed us to appreciate intersecting factors influencing health-seeking in Msitu and Naiti. We present individual factors such as lay etiologies and perceptions of fever, to broader health system barriers such as user fees and drug stock outs which limit access, to socio-cultural determinants at the inter and intra household level. The scalar approach provides us with a more comprehensive picture of the range of different factors that shape an individual's ability to seek out and access remedial actions for febrile illness, discussed in more detail throughout this section.

#### Lay Etiologies of Febrile Illness

In this study, we found that while the interpretation of fever or *homa* (in Swahili) is regarded both as a symptom of illness by a vast majority of respondents, more marginalized respondents (disabled, elderly and those from the poorest households) perceived fever as an illness in itself, which many of these respondents had frequently suffered from. In its basic form, *homa* was described by respondents as a condition straddling the boundaries between sickness and wellness [see also Winch et al. (55)]. As a symptom, *homa* signals the beginning of illness, existing along a continuum from less to more severe. At one end is “ordinary” or “small” fever, *homa ya kawaida* (in Swahili). This is distinguished from “serious/severe” fever, *homa kali* (in Swahili), which is associated with high temperature, severe joint aches and pains, and, in some cases, diarrhea, and vomiting. Respondents often described severe fever as that which prevented them from undertaking their daily responsibilities, such as working on the farm. For example, a young female respondent who was also disabled described severe fever as something that “makes me not to leave the house for days because no one will help me get out as they are afraid of catching it from me” (female FGD participant, Naiti). This young woman, who was physically unable to get out of her wheelchair and who relied on others for assistance, was thus left without care during periods when she suffered from fever. The woman represents specific marginalization at various intersections; gender, disability, economic, and geographical.

At the other end of the febrile spectrum was what was described as “new/unusual” fever, *homa mpya/homa ya siku hizi* (in Swahili), the symptoms of which were often described as resembling that of “un-ending malaria,” that “comes today, goes tomorrow, and returns the next day” (male key informant interviewee, Naiti).

For male respondents, *homa* was something to be expected, and to live with. Men and especially younger men who were typically in herding and animal husbandry roles, believed that they needed to keep going because herd health was more important than taking time off to rest due to suffering a fever. As such, being male and young (aged 40 years or less) was negatively associated with promptly seeking treatment for fever. These findings are consistent with what Mbruru et al. (56) found in a study of lay attitudes of animal to human infections, and how etiologies of febrile illness potentially caused by animal-human interaction influence men and women's treatment-seeking behavior. In our study, and as we describe in detail later, power relations at community and household level shape access to medical resources, including medical fees, money for buying OTC medication, interpretation of illness symptoms or time allocation for gathering herbal treatment from the forest. Moreover, women's age and position in marriage, their relationship with in-laws and broader extended family influenced the interpretation of illness as serious or not and therefore the treatment options that were made available to the patient. For example, illness, including fever, that was believed to result from poor hygiene especially among women of child-bearing age, stigmatized them as these were associated with marital infidelity. In particular, local beliefs around the origin of common infections such as UTIs, which can also cause fever, meant that women, especially younger women would not seek treatment until it is too late.

In both study villages, participants believed that colds and flu represented “ordinary” fever, which did not warrant seeking formalized treatment. Colds and flu were expected to respond effectively to home-based remedies, such as the ingestion of honey mixed with herbs obtained from the forest or *via* buying over the counter drugs such as paracetamol. The expected duration for these kinds of fevers was, for many patients, “no more than 3–4 days” (male FDG participant, Naiti). This category of fever was often cited by respondents as being a normal way the body reacts to stress, a bit like a fever being a way the body fights off illness. The stresses referred to here were associated with everyday living, including bodily responses to tiredness, nutritional changes and fluctuations in local weather conditions. Likewise, Kamat's (57) study of mothers' interpretation of symptoms in their febrile children in Tanzania found that care-seeking was delayed when fever was perceived to be mild and less severe. However, such fever, according to people in Kamat's study, and supported by respondents in our study, could become “serious/severe” fever if left untreated, or it could also develop into “unusual/new” fever which lingered on for weeks and even months, with malaria-like symptoms but worse, and whose treatment could only be accomplished by a doctor trained in Western medicine. This description of unusual fever is of interest because studies in northern Tanzania have found that zoonotic brucellosis can lead to debilitating febrile illness in people, which lingers on in patients if left untreated [see Shirima and Kunda (1), Halliday et al. (2), and Crump et al. (3)].

The distinction between symptoms and illness is conceptualized through three broad understandings, although they vary across the social spectrum, as in the example given above, influenced by social factors such as age and gender, ability, geography, and also by broader limitations on reliable information on febrile illness. Gender, in particular, influenced how men and women understood and categorized febrile illness, and also shaped health-seeking behavior, with men less likely to seek any sort of medical help, while women more likely to ingest honey and herbal remedies.

The first conceptualization is that fever is a common and expected mild health problem. It is ordinary, and little or nothing should be done about it, people should just cope with it. This category of fever was perceived to be the mildest along the continuum of febrile severity. Many respondents indicated that this fever was easily treatable using over the counter drugs or home-based therapies and did not require going to the hospital for treatment. Colds and flu are among illnesses that were associated locally with ordinary fever, and many of those with these symptoms continued carrying out their daily chores such as cultivation, herding and engaging in petty trade. For men, “ordinary fever” did not warrant seeking medical treatment, and they told us that:

“*Women and children complain about normal fevers like malaria, but us men are strong, we only go to the clinic if the fever is pneumonia”* —Men's FGD participant, Naiti.

In general, the health-seeking behavior for this illness was characterized by little or no treatment and observing symptoms over a course of 3–4 days, as this was the duration within which symptoms were expected to subside. If symptoms continued beyond the 4th day, participants perceived this to be serious fever (the second distinction), such as pneumonia, typhoid, a UTI or tuberculosis. Used this way, fever was perceived as a symptom of a serious illness which warranted hospital treatment, especially in women and children.

The third conceptualization of fever as new and unusual was reflected in the way patients talked about the extreme severity of the illness. The concept of “new” was often expressed by respondents who believed this fever to be unusually persistent or chronic and recurring frequently in affected patients. Treatment for both severe and unusual fever was sought from different sources depending on several factors, including gender, age, social economic status, education and physical distance to the nearest health facility, all of which shaped the way severity was linked to particular etiologies. For instance, women tended to associate severe fever with malaria and UTIs, while men associated it with pneumonia, tuberculosis or typhoid perhaps because men spend more time in environments that put them at risk of these infections, for example, herding in the cold, early hours of the morning, eating and drinking contaminated surface water during herding etc. Women's domestic duties such as cleaning and washing with contaminated water may have also exposed them to sanitation and hygiene related infections such as UTIs. Additionally, women perceived unusual fever as resulting from within the environment, caused by a lack of indigenous diets of milk and meat, and changes in soil nutrients that weaken the efficacy of traditional medicine, whereas men believed it to be an external and special illness that was beyond the comprehension of local people, and which could only be treated by Western medicine. To these men, new fever was what Winch et al. (55) describe as a “hospital disease,” or one that was not treatable with home-based remedies. Men in general were more ready to defer to “expertise” of biomedical practitioners, describing them as having “knowledge” on diseases where they themselves were lacking.

Thus, a range of interconnected factors determined how people in this study interpreted febrile symptoms, and the subsequent courses of action needed to treat the fever. Where when and where families sought treatment largely depended on who was sick; child, adult, male or female, married or unmarried, able-bodied or disbaled, household economy and their role in the home unit (herder, mother, husband, etc.) and perception of severity of the illness, as well as other pragmatic factors such as cost, distance, transportation and perceived quality of care.

Interventions aimed at controlling common health problems in communities such as agro-pastoralists, therefore, need to focus on social and cultural stratifiers such as gender, age, and agropastoralists' own notions of health or well-being and what they may mean. In the same vein, our paper focuses on the cultural understandings of febrile illness using broader intersecting lenses, to try and explain why particular behaviors take place, how they play out, who wins and who loses and what might work in providing solutions that are culturally-congruent. In the next section, we look at how social stratifiers intersect with multiple layers of marginalization at the structural, community and household levels, and how this intersection plays out in creating the most enduring barriers to timely health-seeking for febrile illness in agropastoralist settings.

#### Barriers to Health-Seeking in Naiti and Msitu Villages

We found several barriers to health-seeking in Naiti and Msitu villages, and we categorized these into two groups, namely; structural health system barriers and socio-cultural barriers.

##### Health System Barriers

In Naiti village, the informal health sector provided the bulk of healthcare for many families. In most “ordinary” febrile cases, self-treatment with herbal medicine (foraged from forests) as well as self-administered allopathic treatment (medicines are obtained from friends and families or bought from grocery shops as in Figure 2) is commonly used. In other more severe cases of febrile illness, informal providers are consulted. These include unlicensed, and untrained drug sellers (often referred to as *bwana dawa* (or medicineman*)* or *dokta* (doctor) who purchase both allopathic and herbal medicines in bulk from retailers operating in trading centers or pharmacies, and who then move around villages either on bicycles or on foot to sell them in small quantities to their clients. These sellers have specific days and times when they visit the village and they have itinerant or mobile vending operations from where they dispense their medicines. One drug seller operated from a basic “shop” in the village (Figure 2) where he also sold general household merchandise, alcohol and tobacco. VB encountered up to 45 of these mobile drug peddlers in the village between October 2016 and July 2017. They operate outside any regulatory framework, and in many cases they sell prescription-only medication including “antibiotics” (assumed to be penicillin and amoxicillin), which are often sold unlabelled and in clear plastic bags. They also offer diagnostic and therapeutic medical advice to patients and their families.

The second most utilized health providers in Naiti are traditional medical practitioners who mainly operate in exclusive spaces and are consulted in great secrecy. These practitioners primarily utilize non-allopathic modalities such as engaging with spiritual deities to diagnose health problems and herbal medicines for treatment. The authors could not establish the extent to which these providers were utilized for febrile illness or their size, again due to the fact that their operations were mostly discreet.

In Msitu participants also expressed preference to treat themselves for fever informally through sourcing and preparing herbal or lay remedies within the village, yet, unlike those in Naiti, there was also wide reliance on buying biomedical treatments from local drug stores (*duka la dawa*s*)*. When asked what the first remedial action taken to treat fever was in Msitu, almost half (48%, *n* = 90) reported buying medication from one of the two local drug stores in the village. Drug stores in Msitu sold a range of over-the-counter drugs (such as paracetamol) as well as a selection of broad-spectrum antibiotics (such as amoxicillin), and the vendors had some training in dispensing medication. In many cases participants undertook a combination of self-treatment (*via* herbal remedies) in conjunction with biomedical treatments bought from drug stores. Participants often drew on prior experience and knowledge of illness to choose treatment. For example, ingesting Neem tree leaves (*Azadirachta indica*) boiled in water was believed to be a well-known cure for malaria in Msitu and was frequently cited as a treatment when fever sets in.

However, while self-treatment was based on a range of factors reflecting individual knowledge, perceptions and experience with fever, the decision to do so was also rooted in unfavorable conditions within the public health system. Anticipated health system barriers (including user fees, physical inaccessibility, long waiting times, lack of medication, poor staff attitudes and, in some cases, religious bias) were cited as one of the main reasons why people avoid attending health facilities in the event of febrile illness and opt to treat themselves. These barriers are not unique to those in our studies and have been well-acknowledged throughout sub-Saharan Africa [see Leonard (29), Munga and Maestad (31), Gwatkin et al. (34), Mackintosh and Tibandebage (37), Mubyazi et al. (45), and Leonard and Masatu (58)]. Perceptions of medication efficacy and treatment costs were key concerns among participants for why they avoided engaging with health facilities:

“*Consulting a doctor can cost up to 5,000 TSH* (2.16 USD) *but I can come straight here [drug store] and get the same medication for the same price or cheaper.”—*Women's FGD participant, Msitu

“*[I go to the drug store] because of expenses associated with going to hospital and people can't get healed from the medicine they sell there.”—*Women's FGD participant, Msitu

“*There are many challenges. You can get medication from the drug store but don't get better so you go to hospital and they give the same medicine—so you waste money.”—* Men's FGD participant, Msitu

The quotes above highlight the ways in which people make decisions about health care based on their perceptions of the ability of health facilities to cure their condition. In many cases, participants' experiences of the health system fell short of their expectations, leading them to self-treat as a means of saving both time and monetary costs associated with going to hospital.

In addition to perceived adequacy of health services, medicine shortages were a common grievance for both patients and health care practitioners alike. Nurse and doctor interviewee participants often expressed frustration with the Medical Stores Department (MSD), an autonomous branch of the Tanzania government responsible for storing and procuring medication and medical supplies to health facilities. One junior doctor at a referral health facility noted that while they are supposed to be supplied with medication by MSD, continual low stock means that the hospital often needs to source the medication themselves, usually from private firms with premium prices. Furthermore, a nurse at a local health center noted that not only is supply limited throughout the whole country, but in some cases they can be supplied with the wrong medication. Thus, as the health professionals' experience highlights, facility based barriers can often be traced to upstream factors, where logistical constraints limit the supply of medication to hospitals, and thus limit availability to patients. Those in Msitu were cautious about attending health facilities, recognizing that even after they negotiate the various hurdles needed to get there, lack of timely access to medication can result in delayed treatment, prolonging health-seeking and increasing vulnerability.

In some cases, the decision to visit a health facility was heavily influenced by perceptions of religious and tribal discrimination at the Christian-run faith based organizations (FBOs) in the region. In Msitu, two particpants of Muslim faith and Nyaturu heritage felt that other FBOs in the region prioritized Iraqw groups (based on their names) who are predominantly Christian. In this case, they both opted to avoid these facilities in favor of the town hospital where they operated an anonymised numbered system for processing patients, and therefore there was less chance of discrimination.

Finally, costs associated with health facilities also meant that participants often had to spend significant time negotiating ways to secure the cash needed for visiting a health facility, further delaying access to treatment. While all participants in Msitu owned farmland and grew crops, many were unable to sell them for cash due to limited markets:

“*Everyone was harvesting, you can't think of selling your crops because no one has any need of them.”—*Men's FGD participant, Msitu

Inability to sell assets meant that people had to turn to social networks for assistance. Yet this was also fraught with challenges, including fear of burdening others:

“*I didn't tell my daughter [I was sick] because she is already struggling with her children, I didn't want to burden her so I asked a friend for help.”—*Key informant interviewee, Msitu

Fear of inconveniencing others was a common sentiment in borth Msitu and Naiti but perhaps less so in the latter where families approached illness and health collectively. In contrast, in Msitu, respondents rarely reported turning to their social networks for financial assistance, partly due to the perception that no one has the means to help, but also in part due to the idea that social unity does not exist in the same way as it did in the past (i.e., during the sociailst era under President Julius Nyerere). This was particularly prescient for the older populations who often referred to the youths of today as being selfish, as one elder key informant noted: “*they don't love each other*.” Perceptions that people nowadays are more preoccupied with their own needs for survival (due to changed culture around social cohesion and reciprocity) have led to feelings of unease when turning to others for help, with clear material consequences on people's ability to cope during times of illness.

##### Social and Cultural Barriers to Health-Seeking

Health system barriers were not the only considerations in health-seeking decisions in our study. The social and cultural aspects of illness were instrumental in shaping people's choices and access to healthcare. Our findings show that it is the interplay between collective and dynamic approaches to health, the broader social and cultural interpretation of health and ill health in people, animals and the environment, and the gender-based power relations at the household and community levels that play out in decisions regarding seeking treatment in times of illness. Social and cultural attitudes toward health such as lay perceptions and interpretations of health and ill health, ideas regarding quality of healthcare and benefits of treatment options available to patients and their families, threat of illness (perceptions of symptoms and beliefs about susceptibility to and the consequences of the illness), illness etiology and past experiences with similar illness in the family, social networks and the organization of lay health systems, religious beliefs, and, finally, gender differentiation, age and status of the patient and his or her family, all affect health-seeking behavior.

In most cases, the process of health-seeking is ridden with complexities and negotiations. Even where households live close to the health facility and can afford treatment, gender relations (hierarchical power imbalances between men and women) and the dynamics of access and control of household resources, determine whether or not resources are allocated for treatment, where treatment and which illnesses are given treatment priority and which ones are not. We discuss these complexities below.

*Inter/Intra-household Power Relations.* Inter and intra-household power relations involving several wives married to one husband and the extended family affect patients' health-seeking behavior. Reeves and Baden (59) argue that, while conventional economic studies typically sees the household as one “block” where resources are pooled and distributed equally to household members, there is now plenty of anthropological feminist research that highlight that resources are not always pooled, stressing the role of bargaining processes within the household in determining access to resources.

In our study, family size and the extent of “outsider” involvement (in-laws for example) seemed to influence the allocation and distribution of medical resources. For example, in Msitu, households are typically nuclear, comprising a husband and wife and children, and while extended family relations are important, they are less crucial for making decisions around health. Women had greater decision-making power, even where financial costs were involved. In most cases, decisions about where to seek treatments or which health facilities to attend to were made jointly between men and women within the household.

**Figure 5:**
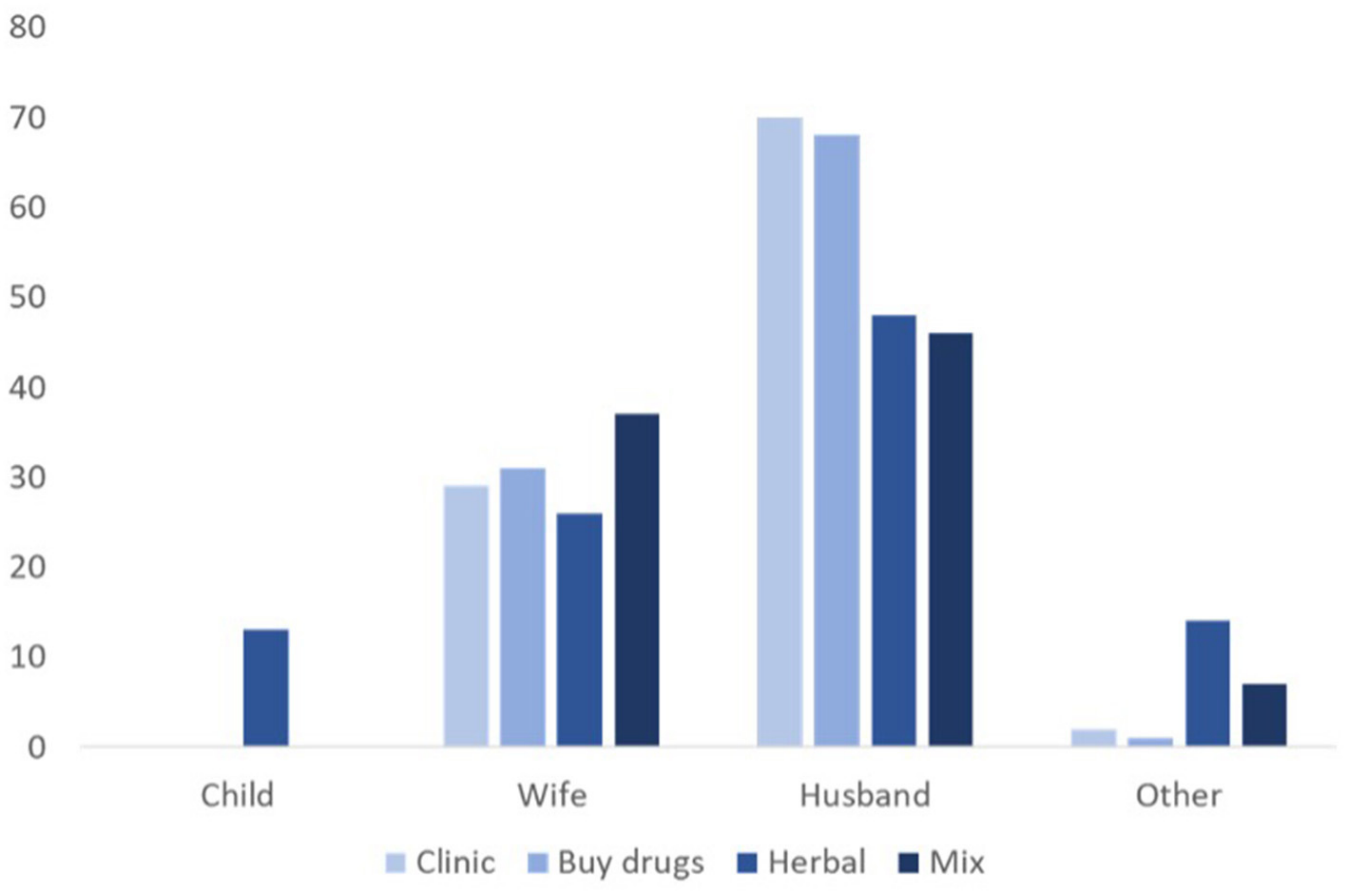
The main decision-maker for household treatment routes in Naiti.

In Naiti, however, whereas there seemed to be cooperation and swift consensus on when, where and how to treat infants when ill, the process was less straightforward for illness amongst adult members of the household. This was complicated by involvement of extended family members in decision-making. To put this into context, in Naiti, an adult man can have various households (in polygamous marriages) that make up one *boma* (extended family). Adult sons and/or their fathers head the *boma* and make decisions for the family, including allocating resources across the households to meet their everyday needs. But allocation of resources in the *boma* is complex and dependent upon the needs, relationships and negotiating power of different family members. This has implications for health outcomes, especially where treatment involves financial resources. Many women that interviewed disclosed that where treatment involved financial costs, men made the decision. Women, however, generally sought treatment earlier and made decisions where treatment was free; for example obtaining herbal remedies from nearby bushes or borrowing medicine from family and friends, and where possible, obtaining cheaper alternative drugs from informal sellers rather than from clinics or pharmacies (Figure 4). As shown in Figure 5, for almost all treatment options available to the household in Naiti, it is husbands (or male relatives if husbands are absent) who make decisions regarding which treatment to pursue. This is especially so when the treatment option is either to visit the clinic or buy drugs from *duka la dawa (*drug store), both of which involve money. Wives are involved in deciding treatment options for themselves or their children when treatment is free, as in herbal therapies, or when relying upon already-stocked pharmaceuticals, either in the household or obtained from neighbors. Women forage for herbal remedies from the forest and mix them with milk or meat soup and blood, before either ingesting them or applying them topically to the affected part of the body. A female febrile patient explained that:

“*When I am ill, I start with what I can personally afford. I have to go to the forest because there is no fee for herbs. But with drugs I need money and my husband may decide to give it or not.” —*Women's FGD participant, Naiti

As is evident from these findings, and well-known in the literature, gender is a strong influence on health-seeking, however, it is also the intersection between gender with the intricate social relationships and the power dynamics within these that give rise to the most pronounced forms of health inequalities. At a structural level, poverty and structural inequalities intersect with social and cultural determinants of health to shape men and women's experiences with healthcare services. A lack of medical resources such as hospital fees, transportation, self-confidence and language barriers etc., can exacerbate women's exclusion from health services as they may be entirely dependent upon male relatives for their health needs. For example, in Ghana, Agbokey et al. (60) and Kuuire et al. (61) found that male family members, particularly husbands, fathers-in-law or adult sons, decided on when and where women sought treatment during illness episodes. Similarly, in Palestine, Majaj et al. (62) found that married women delayed treatment-seeking when ill due to household resource allocation which gave little priority to women's illness. The authors argue that socio-cultural norms prevalent in the villages meant that women were not expected to discuss their illness or express any complaint about it, or to visit a doctor at the first onset of symptoms without having first attempted to solve the problems on their own. This led to severe delays in health-seeking, with negative impacts on women's health outcomes. In Naiti, obtaining a husband's consent, which also means obtaining money for treatment, is crucial to seeking professional healthcare and the reasons for the delays are, as we have shown above, not always financial. They include the need to confirm the nature of the fever (usual, severe, or unusual), to have a positive role in the household and community, and, through social relationships and networks, to reaffirm patients' need to seek treatment and to be healthy.

Children's health, particularly herder boys, is prioritized both by the boys themselves and their families. Often, the treatment routes that are perceived to lead to the fastest recuperation (hospital treatment in this case) are pursued, including ingesting antibiotics obtained from *duka la dawa*, as the boys' role in livestock husbandry is indispensable, and illness in a herder is detrimental to stock welfare. In cases of “ordinary fever,” the boys in particular are expected to manage their illness while still going about their herding duties. Girls, on the other hand, negotiate for treatment through their mothers, which could imply that their health may be neglected where relations between parents are severed.

*The Influence of Lay Referrals and Extended Families.* In Naiti, lay referrals are consulted to identify and label illness, particularly if symptoms are of “unusual fever.” Senior women wield influence when it comes to deciding upon which treatment their daughters-in-law and grandchildren use. They influence their married sons' decisions, particularly when costly treatments such as going to hospital or buying expensive medication are involved. One senior woman, for example, downplayed her daughter-in-law's symptoms and advised the family to use herbal treatments even though both her son and daughter-in-law thought going to the clinic would have been a better option.

**Figure 6:**
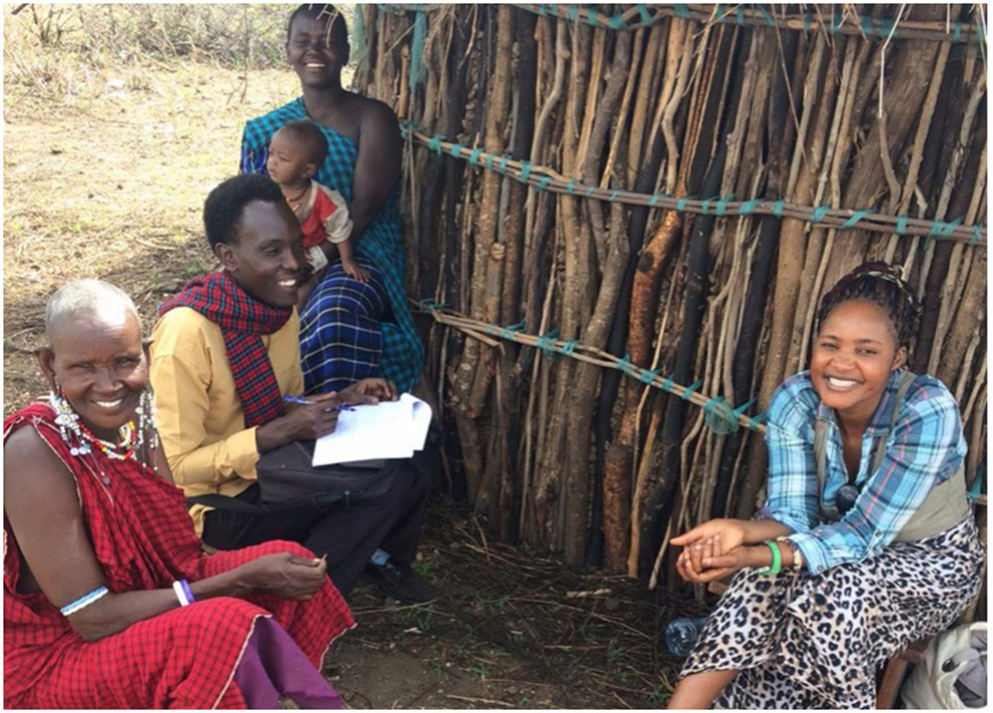
Interview with Naisula and her mother-in-law.

In another case further illustrating these inter- and intra-household power relations, Naisula's 3-year-old son (Figure 6, during my interview with Naisula and her mother-in-law) started having convulsions accompanied with severe vomiting and diarrhea. Worried that her son had “severe fever,” she did not wish to delay his treatment and therefore the young mother decided to go to the village drug seller for advice and treatment. Her husband was not at home at the time and Naisula was not on particularly good terms with her in-laws, which she attributed to her stance against polygamy (she is her husband's only wife), and so she did not consult her extended family about the illness or on the course of action that she was taking. This was in contradiction to social expectations. The drug seller sold Naisula some paracetamol and an unmarked syrup and suggested that she take her son for an injection to cure the convulsions. While still at the shop, Naisula's mother-in-law caught up with her and took the baby away, accusing Naisula of “trying to kill the baby by acting alone in secrecy” (senior woman key informant interviewee, Naiti). By “secrecy,” Naisula's mother-in-law was emphasizing the collective approaches to illness, where diagnosis and decisions regarding treatment are discussed, and advice sought through a process of consultation between younger and older family members. In this case, it would have been Naisula's mother-in-law who would have made the decision regarding where to seek treatment for her grandson, and not Naisula herself. When individuals take these decisions without consultation, their actions are described as secretive and suspicious, and social sanctions are meted out against such individuals, including isolation from communal events such as not being invited to birth/marriage and/or circumcision and death ceremonies. In Naisula's case, her mother-in-law was part of a local therapy management group or lay referral social system in a context where health and ill health are collectively experienced [see also Kleinman (63, 64)], and the young mother, by acting alone, contravened these social norms. The mother-in-law's actions, taking the sick boy away and back to the extended family where the illness was identified and treated, were a social sanction against Naisula for her unexpected behavior. Indeed, the boy was not returned to his mother until a few days later, and when he came back, he had incision marks on his skin, suggesting that a form of traditional healing ritual had taken place. Naisula had to apologize to both her husband and her husband's family for taking the initial decision to treat the boy alone without consulting her wider family network.

Lay referrals in Naiti are composed of older and experienced members of the family such as parents, parents-in-law, traditional healers (when approved of by the *boma*) and more. Patients approach these people, who either collaborate in labeling illness or, where they are unfamiliar with symptoms, as in “new/unusual fever,” advise patients to visit a trained expert, sometimes a medical doctor and sometimes a local healer.

**Figure 7:**
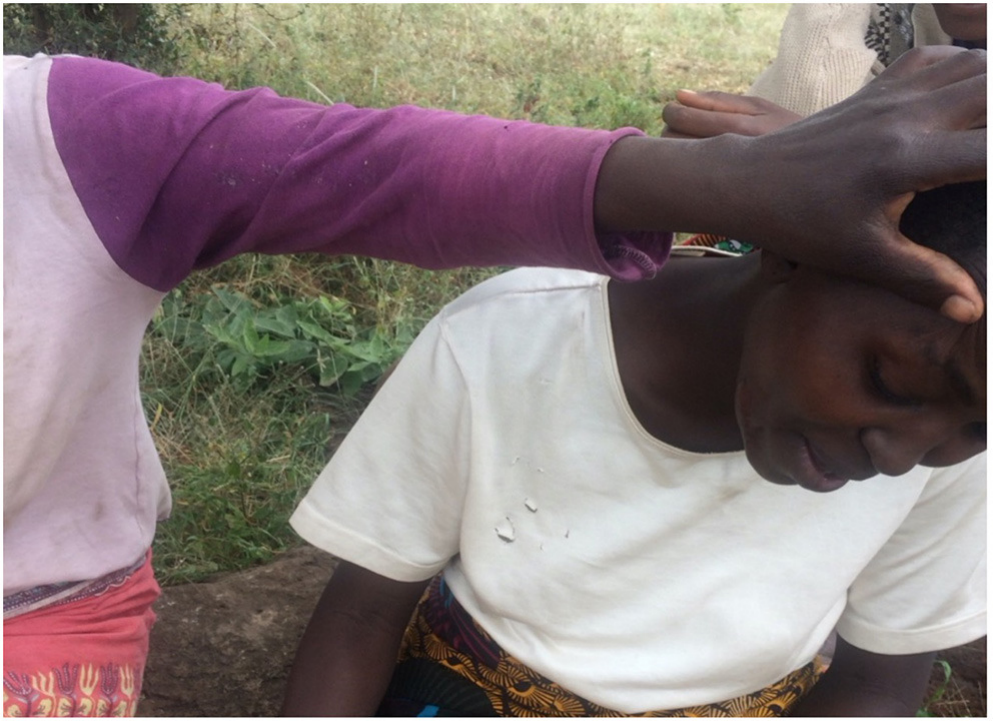
Nasaya being examined for symptoms of illness by local illness referral group.

For the patients and the families that we spoke to, both the exercise of labeling an illness, and the decision to seek treatment (of whichever nature), were based on social norms about the human body and perceptions of what is a normal, healthy body and what is not. As an example, a group of older women came together to help Nasaya, the young mother pictured in Figure 7 to understand and explain her illness. She had complaints ranging from pain in the neck to fever (hot body, chills, headache, and sweating). Since this did not fall into a category of illness that she and her family had experienced before, she had found it necessary to consult those of her female friends who were senior to her in age, and who she therefore regarded as more knowledgeable, to assist with the diagnoses.

After carefully examining her body, Nasaya's friends concluded that she was suffering from “unusual fever,” because her symptoms kept coming back. They also suggested prompt treatment with biomedical drugs and/or a visit to the hospital. But as we illustrated earlier, the decision that Nasaya should seek treatment, and the type of treatment to pursue, would prove more complicated and go beyond the young mother herself and the friends who helped with the diagnosis. As an unmarried woman who had returned to her father's house after her marriage had broken down, she would have to consult her father about the illness label and the recommended treatment, and if it required money then her father would make the final decision as to whether to allocate the money or to advise Nasaya to pursue at-home treatment. Luckily for her, and because the illness fell within the category of “unusual,” her father sold a family goat to raise the money that Nasaya needed to go to hospital in Arusha for treatment. This example again shows the power of labels and how family dynamics and gender factors can influence what actions are taken during illness episodes [see Eyben and Moncrieffe (65) on the power of labeling].

In another example of how health-seeking choices in Naiti are constrained by intra-and extra household power relations, Neema is a young mother and the second wife in a polygamous marriage. She fell ill with what she believed to be *taifodi* fever. She bought a course of what she believed to be amoxicillin antibiotics (because they were not labeled) from a drug peddler, using money she made from selling milk and other small wares at the nearby market. She used these drugs alongside herbal remedies for 4 days, but her symptoms persisted. She had the money necessary to attend the local clinic to be examined by a clinician but could not simply go without first consulting and getting permission from her extended family. Her husband wanted to consult his first wife in advance of Neema making the journey, because this first wife was older and was perceived to be more experienced in interpreting illness symptoms. Moreover, in this case, the husband thought that Neema's condition was improving and thus that she did not need to go to the doctor. Although he perceived the illness to be “severe” fever, he did not believe it to be a “hospital disease” worthy of spending money on [see also Winch et al. (55)]. The first wife shared the husband's view and so Neema's decision to visit the doctor was curtailed.

This example demonstrates how men in Naiti downplay febrile illness and delay treatment for fever unless they perceive it to be “unusual/new” fever (discussed above). In contrast, women generally take “severe” fever seriously and seek treatment earlier because they believe that any delay in health-seeking could lead to symptoms escalating into critical illness that will take them away from their household chores. However, such binary classifications of gender and health only provide part of the health-seeking explanation, because as this example shows, women's journey to seek healthcare is riddled with intersecting layers of barriers, right from the household to the health system itself.

In turning to lay networks for referral during illness, patients are seeking relatable illness experiences, as some expressed:

“*My mother told me that I had the same symptoms as my dead brother, and therefore she advised me to go to the clinic because this was a hospital illness.” —*Women's FGD participant, Naiti

“*When we are ill, we go to my grandfather to tell us what the illness is. He knows many illnesses and their symptoms because he has lived for a long time and he has seen many of the illnesses.” —*Men's FGD participant, Naiti

“*We have a group of people we go to when there is a health problem. If it is ordinary fever you do not need to consult them, but if you have serious [severe] fever, they can tell you which one it is because they are experienced and knowledgeable about illness and treatment.”* Male key informant interviewee, Naiti

“*All of them [senior members in a boma] have experienced this or another illness in their lifetime and they know if you have a similar illness or not. If they tell me to take my child to hospital, I trust them, if they say, this is ok, you can deal with it at home, I also listen to the advice because I trust them.”*—Male participant in youth FGD, Naiti

These examples demonstrate that our study participants are not passive recipients of information and services, who will change their lifestyles in light of health information. Rather, health strategies have to be considered and negotiated with a range of active partners. Although individuals may know what affects their health, depending on their particular position in society (men who reject the need for biomedical healing, junior co-wives who have less power within the *boma*), they can find it difficult to take action as inter- and intra-family and gendered social relationships intersect with health system barriers in multiple and converging ways that result in severe constraints on people's abilities to make choices about their health.

## Discussion: Health Inequalities at the Intersection of Culture and Structure

Throughout this paper we have highlighted the complex intersection between and within barriers at structural (health system) and socio-cultural levels. These multiple and mutating barriers constantly interact in mutable and dynamic ways to limit healthcare choices for specific people in specific places.

McCollum et al. (27) argue that age, gender, geographic location and socio-economic circumstances are key stratifiers, intersecting in mutually-constituting ways to produce vulnerability in health. Geographic location influences people's exposure to environmental risk, access to infrastructural services (roads, clinic services), and political exclusion. Indeed, scarcity of health services in both Naiti and Msitu villages is partly environmental and geographic and partly political. It is environmental in the sense that new emerging fever can be attributed to drastic changes in the environment including longer droughts and shorter rainfall periods (66). It is also political as poor infrastructure typically in pastoralist settings is a reflection of pastoralists' relative lack of power, living in geographic areas marginal to the national political process. Gender and sex leads men and women to experience febrile illness challenges very differently. Biologically, women's bodies experience health challenges in unique ways than do men, and socially, gender is constituted through a wide range of norms and gendered power relations which exclude women from decision-making while excluding others (generally men) from domains such as the health clinic. Age too is significant because “social vulnerabilities emerge at different life stages through intersection with other social forces” [(27), p. 21]. Age intersects with gender and patriarchy to marginalize young girls in different ways to those affecting women or elderly men. And finally, socio-economic situations are significant because the lack of resources limit individuals' decision-making power and choices. These intersecting factors are both multi-scalar, operating across national, local, household and individual levels to shape peoples' everyday experiences, and temporal (67) in relation to age, seasons, biological rhythms and health.

Moreover, within resource-limited pastoral and agro-pastoralist settings, approaches to risk are shaped by broader socio-economic and livelihood challenges intersecting with socio-cultural factors that make life possible, yet also amplify vulnerabilities for specific categories of people. For example, people who do not own resources and cannot work—such as elderly men or disabled community members—have extremely limited choices. Schneider (68) cautions that public health interventions to reduce public health risks ought to be designed using culturally appropriate mechanisms that target specific forms of inequalities as well as challenge the entrenched power relations that create these inequalities. Finding ways to do this in places like Naiti or Msitu, where socio-economic deprivation intersects with geography, gender and age to produce extreme marginalization for particular, isolated individuals is especially challenging.

While some health research tends to suggest that lay behavior and motivations behind treatment-seeking decisions depend on the value people place on the benefits of taking a specific action to respond to ill health (69), it also assumes that people have choices available and that they can weigh the cost and benefit of each. However, though this may be the case in some contexts, in Naiti and Msitu, healthcare options are limited, which means that people often use the most feasible option available to treat illness regardless of the outcome or perceived benefits (or lack thereof) of the chosen route of treatment. The care-seeker in this case, as Good (70) observes in other contexts, is not ultimately free to make voluntary choices as is implied.

In Msitu, health system factors heavily influenced decisions on where and when to seek treatment for fever. Participants painted a bleak picture of the public health system, where religious bias, lack of medication, long waiting times and treatment costs render health facilities as places to be avoided during illness, rather than places where they can be healed. As we argued above, the convergence of political and geographical marginalization here is reflected in health system deficits. As such, participants opted to treat themselves either through local remedies or *via* buying medication from local drug stores. Financial barriers were one of the biggest deterrents for accessing health care. People had to spend significant time negotiating resources to secure the cash needed to visit a health facility, however, as our findings show, this step is often fraught with challenges. Limited options to sell assets to secure cash, precarious social relations and lack of social safety nets influence the extent to which people can draw on these resources, and thus their ability to proceed with health-seeking or to abandon the process altogether. Perceptions of medication effectiveness, medicine stock outs and religious bias within health facilities also influenced decisions on health-seeking. Thus, while places like Msitu have health facilities located within relatively close proximity to the village, there are a range of interconnecting structural and institutional barriers which determine how and when individuals can seek remedial actions for febrile illness.

Women's health-seeking in particular is also further curtailed by the gender power relations within and between households. As is evident in the example of Neema, her gender intersected with her age, perceived experience in labeling illness as well as her relationship with her co-wives, which all contributed in determining her husband's willingness to support and allocate resources for treatment, and this has implications for when illness is detected, and in whom. One of the subsequent implications is that treatment is delayed, and “severe” fever may not be addressed until it is too late, even for relatively wealthy household members, because of these complexities. Clearly, in this situation, even if Neema had been able to weigh up the costs and benefits of available options and determined she would benefit from visiting a doctor and could afford the cost of treatment, she was unable to act on her choice. This was because she was not the main decision maker and, ultimately, she had little influence on the decision to seek healthcare. As Poortaghi et al. (71) have argued, an awareness of these factors is imperative to the planning process for successful interventions and the expansion of existing health services in any setting.

Health-seeking is about aspiring to have an acceptable level of well-being based on the awareness of the health problem and cooperation on the part of patients, their families and local health systems (32, 68, 71). It is an approach through which people can monitor their bodies or, as Turner (72) added, their social contexts, to distinguish between symptoms and interpret them, to look for medical interventions and apply other supportive resources when deemed necessary. In Naiti and Msitu, residents need to engage with social networks based around family and kin to collectively interpret and label health problems. Community elders (both male and female) who enjoy significant social power often explain symptoms and label illness within an existing socio-cultural framework. Elders do not always have the final say in relation to an illness diagnosis, as there is great emic diversity in the way people act in response to febrile illness. They do however, influence what treatment is pursued, especially if it involves financial costs. For example, where family members disagree over illness symptoms, an experienced elder in the family is consulted to interpret symptoms and recommend the most appropriate treatment. During the course of our study, there were situations where patients thought they had a “severe fever,” but elders downplayed it to “ordinary fever” and recommended herbal remedies.

Sometimes, as we noted earlier, younger people, especially boys, were more likely to prioritize hospital treatment for severe febrile illness over at-home remedies. The boys perceived this treatment as aiding faster recuperation, to in turn enable them to resume their very important herding chores. Men in general, and younger men in particular, are exposed to urban settings, where they interact with a diverse selection of therapies, as opposed to older cohorts of the population who often rely on traditional herbal remedies, which they were also most familiar with. For example, young people (mostly men) tended to associate pharmaceutical drugs and/or going to the hospital with being “modern.” Therefore, delays in presenting at formal health facilities, and early detection of potentially life-threatening illness, could be a bigger problem for older people in our study settings, as well as women of all ages, than among the younger male population.

This ambiguity around healing is also reflected in other forms of treatment. As already noted, the informal health sector, characterized by unlicensed drug peddlers who diagnose both human and livestock illness, is dominant. Naiti's residents seek “expert” advice when treatments are ineffectual or when illness symptoms become severe. “Experts” include anyone who sells drugs, such as unlicensed peddlers, shopkeepers and pharmacists, both inside and outside the village. For the latter, consultation is usually made by mobile phone: once symptoms have been described, the drug seller offers a presumptive diagnosis and suggests a prescription. In most cases, the suggested therapies are then borrowed from extended family members that stock them. Sharing medicine (both traditional and allopathic) is a fairly common practice in the villages, and whenever there is a severe illness episode, in the first instance, patients' families consult kin and neighbors for medicines. Ultimately, the decision to consult others in illness diagnoses rested on the patients' (and/or their carers') familiarity with a set of historically acceptable symbols or symptoms signifying a particular illness, such as cold chills and muscle pain for perceived malaria, or wheezing and shortness of breath for pneumonia

For this reason, assumptions regarding how people can be expected to behave with regards to health-seeking in places like Naiti and Msitu cannot be easily made due to the intersecting barriers to health-seeking, as well as the plural and collective experiences of illness that go beyond a single episode of illness, and beyond an individual patient. It is therefore important for researchers to explore these dynamics, and the value of innovative approaches such as intersectionality in arriving at conclusions cannot be overemphasized.

## Conclusion: What Works to Reduce Barriers to Health-Seeking in Agro-Pastoralist Settings?

Public health control measures emphasize behavioral change, such as appropriate use of drugs, improved diagnostic capacity and technical solutions, as ways to reduce transmission of diseases, without considering the complexities and dynamic processes involved in health-seeking in agro-pastoralist communities living in remote, hard-to-reach settings. As we show in this paper, biomedical approaches would have limited impact in our study area where illness is also experienced collectively and socially. People rely on cultural health beliefs that influence how symptoms are perceived based both on the physical (symptom categories), personal (patient profile), historical (experience of previous illness), and social (socio-cultural) contexts within which illness occurs. Some people may choose to live with rather than seek to ameliorate symptoms if they or others in their social network believe that the symptoms do not constitute a serious health problem warranting medical attention. But where symptoms are believed to be serious and a cause for concern, response and actions are influenced by the immediate material conditions that the patient and his or her family find themselves in. Lay referral networks may put pressure on a patient to seek remedies (based on perception and interpretation of the illness) and help with mobilizing resources for the patient, to seek professional treatment, or even share past illness experiences upon which treatment can be based. Decisions to approach family and friends or engage in self-treatment or visit a clinic are further influenced by inter- and intra-household relationships and interests. Whether people treat themselves or consult family, friends or medical services is complicated further by other factors such as support for health-seeking, ability to secure financial resources, and access to, availability of and quality of healthcare.

Ultimately, people's decisions to engage with a given medical channel are influenced by many factors, including the type of illness and perceptions about its severity, who it affects within the household, access to what therapies and for whom, and the perceived quality of the service, as well as financial and infrastructural constraints and familial or household dynamics.

In our study villages, in some instances, residents may choose traditional healers and untrained village allopathic *doktas* above formally trained practitioners or government health facilities due to the mistrust of the health system, for reasons we discussed above. In other cases, and upon consultation with lay referral groups comprising family and kin, they may decide that a particularly severe or new type of illness warrants professional and costly treatment. In all instances, gender intersects with household power dynamics, social relationships, and the availability of income, which can and often do complicate decisions about healthcare options. Neither route necessarily offers a clear-cut trajectory back to health or a dependable means of dealing with disease.

In conclusion, and in answer to the question, what works to reduce sex and gender disparities in health and disease, we argue that disease interventions centering on the individual rather than the collective are unlikely to work agro-pastoralist settings, where health disparities occur in collective, mutable and intersecting levels. We believe that it is crucial to explore and understand how structural, social and cultural complexities such as household and intra-household relationships impact care-seeking and health outcomes in agro-pastoralist settings where illness is collectively experienced and where treatment is defined by these processes. Furthermore, we draw attention to the structural factors or health system barriers, which play a role in determining decisions to engage with health facilities in the event of illness. Appreciating these interconnecting factors can lead to improvements in the design of health interventions, which could result in greater uptake by targeted populations.

Methodological approaches suited for uncovering these complexities include in depth, long-term ethnographic work, in addition to participatory approaches which can help to uncover differential experiences of health and illness. As our work shows, when considering health interventions, the voices and experiences of those who are the “object” of interventions should be prioritized and heard, in order to co-create health programmes that are appropriate for context and sustainable in the long term. Moreover, given the importance of community dynamics and reliance on community-level health providers (through *doktas, duka la dawas* or referral networks) we suggest health care at the local level as a key focus for broader health system strengthening.

## Data Availability Statement

The raw data supporting the conclusions of this article will be made available by the authors, without undue reservation.

## Ethics Statement

All participants provided written or verbal informed consent. The study was approved by the Ethics Review Committees of the Kilimanjaro Christian Medical Center and National Institute of Medical Research in Tanzania, and in the UK by the Ethics Review Committees of the College of Medical, Veterinary and Life Sciences at the University of Glasgow and the Institute of Development Studies at the University of Sussex. Approval for study activities was also provided by the Tanzanian Commission for Science and Technology (COSTECH) and by the Tanzania Wildlife Research Institute (TAWIRI) as well as by regional, district, ward, and village-level authorities in the study area. All individual names, including any identifying information have been changed in order to preserve anonymity. Some place names have also been changed, except in mobile pastoralist study sites with no particular territorial boundaries. Written informed consent was obtained from the individual(s) for the publication of any potentially identifiable images or data included in this article.

## Author Contributions

VB and JV contributed to conception and design of the study. VB wrote the first draft of the manuscript. JV wrote sections of the manuscript. All authors performed analysis, contributed to manuscript revision, read, and approved the submitted version.

## Funding

This research was funded by the Biotechnology and Biological Sciences Research Council, Department for International Development, the Economic and Social Research Council, the Medical Research Council, the Natural Environment Research Council and the Defense Science and Technology Laboratory, under the Zoonoses and Emerging Livestock Systems Associated Studentships (ZELS-AS) programme (BB/N503563/1). The funders had no role in study design, data collection and analysis, decision to publish, or preparation of the manuscript.

## Conflict of Interest

The authors declare that the research was conducted in the absence of any commercial or financial relationships that could be construed as a potential conflict of interest.

## Publisher's Note

All claims expressed in this article are solely those of the authors and do not necessarily represent those of their affiliated organizations, or those of the publisher, the editors and the reviewers. Any product that may be evaluated in this article, or claim that may be made by its manufacturer, is not guaranteed or endorsed by the publisher.
